# MicroRNA-210 regulates the metabolic and inflammatory status of primary human astrocytes

**DOI:** 10.1186/s12974-021-02373-y

**Published:** 2022-01-06

**Authors:** Nicholas W. Kieran, Rahul Suresh, Marie-France Dorion, Adam MacDonald, Manon Blain, Dingke Wen, Shih-Chieh Fuh, Fari Ryan, Roberto J. Diaz, Jo Anne Stratton, Samuel K. Ludwin, Joshua A. Sonnen, Jack Antel, Luke M. Healy

**Affiliations:** 1 Neuroimmunology Unit, Montreal Neurological Institute, McGill University, Montreal, QC Canada; 2 Department of Neurology and Neurosurgery, Montreal Neurological Institute, McGill University, Montreal, QC Canada; 3 Centre for Research in Neuroscience, The Research Institute of the McGill University Health Center, Montreal, QC Canada; 4 Department of Pathology, Queen’s University, Kingston, ON Canada; 5 Departments of Pathology, Neurology and Neurosurgery, McGill University, Montreal, QC Canada

**Keywords:** Astrocyte, Stroke, MicroRNA-210, Ischemia, Hypoxia, Inflammation, Multiple sclerosis

## Abstract

**Background:**

Astrocytes are the most numerous glial cell type with important roles in maintaining homeostasis and responding to diseases in the brain. Astrocyte function is subject to modulation by microRNAs (miRs), which are short nucleotide strands that regulate protein expression in a post-transcriptional manner. Understanding the miR expression profile of astrocytes in disease settings provides insight into the cellular stresses present in the microenvironment and may uncover pathways of therapeutic interest.

**Methods:**

Laser-capture microdissection was used to isolate human astrocytes surrounding stroke lesions and those from neurological control tissue. Astrocytic miR expression profiles were examined using quantitative reverse transcription polymerase chain reaction (RT-qPCR). Primary human fetal astrocytes were cultured under in vitro stress conditions and transfection of a miR mimic was used to better understand how altered levels of miR-210 affect astrocyte function. The astrocytic response to stress was studied using qPCR, enzyme-linked immunosorbent assays (ELISAs), measurement of released lactate, and Seahorse.

**Results:**

Here, we measured miR expression levels in astrocytes around human ischemic stroke lesions and observed differential expression of miR-210 in chronic stroke astrocytes compared to astrocytes from neurological control tissue. We also identified increased expression of miR-210 in mouse white matter tissue around middle cerebral artery occlusion (MCAO) brain lesions. We aimed to understand the role of miR-210 in primary human fetal astrocytes by developing an in vitro assay of hypoxic, metabolic, and inflammatory stresses*.* A combination of hypoxic and inflammatory stresses was observed to upregulate miR-210 expression. Transfection with miR-210-mimic (210M) increased glycolysis, enhanced lactate export, and promoted an anti-inflammatory transcriptional and translational signature in astrocytes. Additionally, 210M transfection resulted in decreased expression of complement 3 (*C3*) and semaphorin 5b* (Sema5b)*.

**Conclusions:**

We conclude that miR-210 expression in human astrocytes is modulated in response to ischemic stroke disease and under in vitro stress conditions, supporting a role for miR-210 in the astrocytic response to disease conditions. Further, the anti-inflammatory and pro-glycolytic impact of miR-210 on astrocytes makes it a potential candidate for further research as a neuroprotective agent.

**Supplementary Information:**

The online version contains supplementary material available at 10.1186/s12974-021-02373-y.

## Background

Astrocytes contribute to central nervous system (CNS) homeostasis through their critical roles in blood brain barrier (BBB) maintenance, production of pro- and anti-inflammatory cytokines, release of antioxidants, and provision of trophic factors to surrounding neurons and oligodendrocytes [8, 37, 42, 53, 56]. Astrocytes can contribute to the pathology of multiple neurological disorders, either through the loss of their physiologic functions and/or the production of disease-associated molecules [12, 15, 44, 45, 50, 60, 62]. Conversely, in the context of disease astrocytes can provide tissue protection and support repair through the export of lactate, which provides neurons with an additional energy source, and through the release of anti-inflammatory/neuroprotective factors that promote neuronal regeneration and survival [13, 32].

The dynamic properties of astrocytes in health and disease are partially regulated by their microRNA (miR) expression profile. miRs are ~ 22 nucleotide strands of RNA that post-transcriptionally regulate protein expression [51]. Previously, we used laser-capture microdissection (LCM) to report the miR profile of astrocytes in human adult white matter (WM) differs from that of astrocytes in the gray matter (GM) [49]. Our follow-up study showed a distinctive miR expression profile of astrocytes around multiple sclerosis (MS) lesions compared to astrocytes from normal appearing tissue [48]. Of interest was our finding that miR-210 expression was increased in astrocytes around active MS lesions [48]. miR-210 has previously been associated with ischemia and is reportedly increased in the mouse brain subjected to the middle cerebral artery occlusion model of stroke [21]. In combination, these results are consistent with the concept of ongoing ischemic stress in MS lesions [1, 33]. However, neither the expression of miR-210 in human brain tissue undergoing confirmed ischemic injury nor its functional role in glial cells have been evaluated thus far.

In this study, we determined the miR expression profile of astrocytes around human ischemic stroke lesions and compared this to the profile of astrocytes isolated from neurologic control tissue. We uncovered increased miR-210 expression in WM astrocytes responding to ischemic stress in-situ. To understand the functional effect of increased miR-210 expression in human astrocytes, we developed an in vitro stress assay using primary human astrocytes derived from the fetal brain and investigated miR-210-mediated modulation of human fetal astrocyte (1) metabolism, (2) inflammatory cytokine production, and (3) production of potentially neuroprotective molecules. Specifically, we measured oxygen consumption rate, glycolysis, lactate export, pro- and anti-inflammatory cytokine expression, and the expression of genes previously reported to have neurotoxic/neuroprotective roles. We show for the first time that miR-210 is upregulated in astrocytes around stroke lesions, and that miR-210 mimic promotes astrocytic lactate export while decreasing inflammatory cytokine production.

## Methods

### Neuropathological identification of infarcts in human brain tissue

Sixteen adult human brains specimens aged 30–93 were obtained from the Montreal Neurological Institute and Hospital (Montréal, Québec, Canada) and Kingston General Hospital (Kingston, Ontario, Canada). Formalin-fixed paraffin-embedded (FFPE) sections of cerebrum were evaluated by a board-certified neuropathologist (J.S.) using established criteria [35]. Briefly, Hematoxylin and Eosin-stained sections were assessed for the presence of hyper-eosinophilic (acutely hypoxic) neurons, infiltrating neutrophils, alteration of parenchyma (rarefaction or cavitation), loss of hypoxia sensitive cells (neurons in GM and oligodendrocytes in WM), gliosis, mononuclear cell response (microgliosis or foamy macrophages), active angiogenesis and hemosiderin deposition in scavenger cells. Acute infarcts were primarily characterised by the presence of hyper-eosinophilic neurons with rarefaction or early cavitation, while chronic infarcts all demonstrated well developed gliosis and advanced cavitation. Tissues characterised as neurological controls contained occasional hemosiderin deposition but no other changes.

### Immunohistochemistry and laser-capture microdissection

Laser-capture microdissection (LCM) was performed as previously described [49]. Briefly, FFPE sections were stained with an anti-GFAP (glial fibrillary acidic protein, Ventana cat. # 760-4345, Cell Marque, CA) antibody for astrocyte identification and cell capture. Cells were visualized using 3,3’-diaminobenzidine (DAB, Vector Laboratories, USA, SK-4100) and a secondary antibody conjugated to horse radish peroxidase (HRP). For stroke samples, GFAP-positive astrocytes located around lesions, avoiding cells inside or distal from the lesion itself, were identified and captured using a PALM-LCM. All cells were captured from cortical gray matter or the adjacent subcortical white matter. Approximately 35 cells were captured from each slide, after which the cells were lysed in TRIzol and stored at -80 °C until further use. For slides with both WM and GM stroke lesions, captures were performed from both regions.

### RNA extraction and quantitative polymerase chain reaction for LCM

RNA extraction and quantitative reverse transcription polymerase chain reaction (RT-qPCR) were carried out as described previously [49]. Briefly, RNA was extracted using a standard TRIzol protocol. The miR profile chosen for investigation was based on a previous study that identified miRs which were (1) expressed by astrocytes and (2) had predicted associations with neurodegenerative diseases [48]. Reverse transcription was performed with Taqman primers specific to each miR of interest (ThermoFisher, USA). A pre-amplification step was performed on the complementary DNA (cDNA) prior to RT-qPCR [49]. RT-qPCR with Taqman probes was then used to measure the expression of each miR of interest. Data is presented relative to the mean cycle threshold (CT) value of all miRs measured, which has previously been shown to be an effective method of normalization [36]. CT values > 37 were discounted. Astrocytes captured from the WM around stroke lesions were compared to astrocytes captured from neurologic control WM cells, while those from GM stroke lesions were compared to cells from neurologic control GM astrocytes. For tissue sections with astrocytes captured from both the WM and GM, the WM cells were captured and pooled first, and then a new tube was used to collect and pool all GM cells. Therefore, the two cell populations were not combined. −∆∆CT was calculated using the previously defined method [31], and fold change was calculated as 2^−∆∆CT^.

### RNA extraction and RT-qPCR for in vitro samples

RNA extraction and reverse transcription of in vitro samples were performed as described above, with the modification that random hexaprimers were used instead of specific primers during the reverse transcription [20]. RT-qPCR was performed with Taqman probes for each gene or miR. Messenger RNA (mRNA) CT values were normalized to glyceraldehyde 3-phosphate dehydrogenase (*GAPDH)*, while miR CT values were normalized to U47, a previously defined, stable miR [18].

### Culturing of primary human astrocytes and HeLa cells

Primary human fetal astrocytes were isolated from 2nd trimester fetal tissue (17–23 weeks of gestation) obtained from the University of Washington birth defects research laboratory (BDRL, project#5R24HD000836-51). As previously described [20], fetal CNS tissue was chemically dissociated with 0.05% trypsin (ThermoFisher) and 50 µg/mL DNase I (Roche) and was subsequently mechanically dissociated with scalpels and washed with room temperature phosphate buffered saline (PBS) containing 100 units/mL penicillin–streptomycin (PS, ThermoFisher, USA, 15140-122) and 500 ng/mL Amphotericin B (ThermoFisher, USA, 15290-018). Cells were plated on 10 µg/mL poly-L-lysine (Sigma, USA, P8920) coated flasks at 3 × 10^6^ cells/mL in T75 flasks using DMEM/F12 (ThermoFisher, USA, 11320033) with 10% fetal bovine serum (FBS, Wisent Bio Products, Canada, 080150), 1% PS, and 1% Glutamax (ThermoFisher, USA, 35050-061) and grown in incubators set to 37 °C with 5% CO_2_. For in vitro stress conditions, astrocyte media was aspirated, cells were washed with 37 °C PBS, and the fresh, appropriate media warmed to 37 °C was then added into wells. The cells were treated for 24 h with 1 ng/mL interleukin-1beta (IL1b, Gibco, PHC0815) in basal media, glucose-free DMEM/F12 media (ThermoFisher, USA, 11966025), or 1% O_2_ in normal culture media for inflammatory, metabolic, and hypoxic stress, respectively. For the control condition, the media aspiration and PBS washing occurred alongside all treated wells, and fresh normal culture media was added for 24 h before collection.

### Culturing of primary human microglia and oligodendrocytes

Human oligodendrocytes and microglia were isolated as previously described [9, 11]. Briefly, post-natal human brain tissues were obtained from non-malignant cases of temporal lobe epilepsy, digested by 0.5% trypsin (Thermofisher) and 25 µg/mL DNase (Roche) treatment, and mechanically dissociated through a nylon mesh filter. Tissue homogenate was then subjected to Percoll (Sigma-Aldrich) gradient centrifugation to isolate the glial cells. Further purification of microglia was performed through magnetic-activated bead sorting of CD11b + cells (Miltenyi Biotec). Remaining oligodendrocytes were plated on chamber slides or 12-well plates (ThermoFisher) coated with poly-L-lysine and extracellular matrix (Sigma-Aldrich). Microglia were maintained in Minimum essential medium (MEM) with 5% FBS and 1% PS. Human oligodendrocytes were cultured with DMEM/F12 supplemented with N1 (Sigma, Canada, N6530), 0.01% bovine serum albumin, 1% PS, B27 supplement (ThermoFisher, 17504044), platelet-derived growth factor (PDGF-AA, 10 ng/mL), basic fibroblast growth factor (bFGF, 10 ng/mL), and triiodothyronine (T3, 2 nM, Sigma). Both microglia and oligodendrocytes were cultured at 37 °C under a 5% CO_2_ atmosphere.

### Assessment of astrocyte purity by immunocytochemistry

Cells on a chamber slide were incubated with anti-O4 antibodies (1:200; #MAB345, Sigma-Aldrich) for 5 min and fixed in 4% paraformaldehyde solution for 15 min at 37 °C. Following permeabilization with PBS containing 0.2% Triton X-100 and 3% goat serum for 1 h, cells were incubated with Cy3-conjugated anti-GFAP (1:200; C-9205, Sigma-Aldrich) and anti-PU.1 antibodies (1:500; 2258S, Cell Signalling) at 4 °C. The next day, cells were stained using Alexa Fluor 488-conjugated anti-mouse IgM antibodies (1:500; A21042, Invitrogen), Alexa Fluor 647-conjugated anti-rabbit antibodies (1:500; A27040, Invitrogen) and Hoechst 33342 (1 mg/mL; Invitrogen). Percentage of cells positive for GFAP, O4 or IBA1 staining in astrocyte culture was quantified using a CellInsight CX7 High Content Screening Platform (ThermoFisher). All conditions were assessed in triplicate.

### HeLa cell culturing, treatment, and collection

HeLa cells were cultured using DMEM/F12 with 10% FBS, 1% PS, 1% Glutamax warmed to 37 °C. Once the cells were 70% confluent, supernatant was aspirated, cells were washed with 37 °C PBS, and fresh media was added. Cells were then put into the hypoxia chamber at 1% O_2_ for 2–48 h or kept in the normal incubator for the control condition. For protein collection, cells were removed from the hypoxia chamber, and cells were lysed by adding 200 µL RIPA buffer to each well of a 6-well plate and scraping within 1 min of being removed from the hypoxia chamber. The cell lysates were gently rotated for 1 h, after which they were spun down at 3000RPM for 15 min in a tabletop centrifuge. The supernatant was transferred to a fresh Eppendorf tube and the pellet was discarded. The remaining cell lysate was then stored at − 80 °C before use for Western blot.

### Transfection of primary human astrocytes

Lipofectamine RNAiMax (ThermoFisher, USA, Cat# 13778075) was used for Alexa Fluor 555 BLOCK-iT control (ThermoFisher, USA, Cat# 14750-100) and for miR-mimic/seed transfections following the manufacturer’s protocol. miR-210-mimic (210M) was created by annealing miR-210-3p and miR-210-5p strands, with the miR-210-3p strand as the functional strand. To generate the seed mutant (miR-210-seed, 210S) for use as highly specific control, the seed regions were transposed between the miR-210-3p and miR-210-5p strands, which has been shown to reduce the binding ability of miRs to their targets [17]. The normal 3p and 5p nucleotide sequences were CUGUGCGUGUGACAGCGGCUGA and AGCCCCUGCCCACCGCACACUG, respectively, whereas the mutated 3p and 5p sequences for use in the 210S construct were CACACGCUGUGACAGCGGCUGA and AGCCCCUGCCCACGCGUGUCUG, respectively. The two strands of the mimic and of the seed-mutant were obtained from Integrated DNA Technologies (IDT) using the publicly available sequence of miR-210. RNA strands were annealed following the IDT protocol for annealing oligonucleotides. Cells were transfected with 210M and 210S at a final concentration of 50 nM in normal culturing media after the microRNA was incubated with Lipofectamine RNAiMax in serum-free media for 10 min. For cells treated in hypoxic and inflammatory conditions after transfection with 210M or 210S, cells were washed with 37 °C PBS 48 h following transfection, and then transferred into the appropriate stress condition.

### Middle cerebral artery occlusion (MCAO) surgery and tissue preparation

Permanent focal cerebral ischemia was induced using middle cerebral artery occlusion (MCAO) as described previously [61]. In brief, male C57BL/6 mice, 8–12 weeks of age, were kept anesthetized with isoflurane in O_2_ (0.5–1 L/min; inhalation). During surgery, body temperature was maintained at 37.0 ± 0.5 °C using a homeothermic system with a rectal probe (Harvard apparatus). The trunk of the left middle cerebral artery (MCA) was exposed by making a small temporal craniotomy and then ligated just before its bifurcation between the frontal and parietal branches with a 9-0 suture. Visual inspection under an operating microscope was performed to confirm the complete interruption of blood flow. In addition, the left common carotid artery was occluded. After surgery, animals were returned to their cages with free access to water and food. Fourteen days after surgery, mice were perfused with PBS followed by 4% paraformaldehyde in 0.1 M phosphate buffer. After post-fixation in the same fixative for 24 h, the tissues were cryoprotected by immersing in 30% sucrose in PBS. Cryostat coronal section (14 μm thick) of the brain were used for the immunofluorescence labeling.

### microRNAscope (miRscope)

The miRscope assay was performed using an Advanced Cell Diagnostics (ACD) kit (ACD, USA, miRNAscope™ HD (Red) Assay, UM 324510) to visualize miR-210 in mouse tissue. The kit was used following manufacturer’s instructions for fixed frozen mouse tissue. In brief, tissue was incubated in target retrieval solution for five minutes at 95 °C. Sections were then washed with 100% EtOH and air-dried for five minutes at room temperature. Sections were surrounded using a hydrophobic barrier pen (ImmEdge, Vector Labs, USA, 310018) and then incubated with protease III provided in the ACD kit for 20 min at room temperature. Sections were washed once with PBS before following the multiplex steps as described in the protocol. All incubations were at 40 °C and were performed in a humidity chamber (HybEZ oven, ACD, USA, 321711). miR-210 was visualized in tissue using a miR-210 probe (ACD, USA, 728551-S1). Nuclei were stained with DAPI (1/5000, Invitrogen). Slides were mounted using PermaFluor Mounting Media (Thermofisher). Images were obtained using a Zeiss Axio Observer. miR-210 positive nuclei were counted by eye. Cells with at least one punctum in the nucleus were counted as one positive cell, while cells without nuclear puncta were counted as negative cells. 300–500 cells were counted for each region per slide.

### Seahorse analysis

Seahorse bioanalysis was carried out to measure the oxygen consumption rate (OCR) and extracellular acidification rate (ECAR) of primary human fetal astrocytes, a proxy measure for cellular oxidative phosphorylation and glycolysis, respectively [55]. Primary human astrocytes were plated on uncoated XFe-96 plates 48 h before analysis at a density of 15,000 cells per well. The standard Seahorse guide was followed, using three sets of mixes, zero sets of waits, and three sets of measuring after each injection, as is described in the Seahorse manual. Basal Seahorse media (Agilent, USA, 102353) with the addition of glucose (17.5 mM), glutamine (2 mM), and pyruvate (1 mM) was used for OCR analysis. The media was only supplemented with glutamine (2 mM) for ECAR analysis. The pH of both medias was adjusted to 7.4 after the addition of the substrates. Oligomycin, FCCP, and Rotenone/antimycin-A (Agilent, USA, 103015) were used for OCR analysis at final concentrations of 1 µM, 1 µM, and 0.5 µM, respectively. Glucose, oligomycin, and 2-deoxyglucose (Sigma, USA, D6134) were used for ECAR analysis at final concentrations of 10 mM, 1 µM, and 50 mM, respectively.

### Western blots

Cell lysates were obtained using RIPA lysis buffer (Solution made to match Rockland, USA, MB-030-0050, with addition of Roche, Switzerland, 04693116001 protease inhibitor and the Roche, Switzerland, 04906837001, PhosStop phosphatase inhibitor). Proteins were separated by SDS-PAGE on 10% gels and transferred to nitrocellulose membranes using a semi-dry transfer apparatus (Bio-Rad, Hercules, CA, USA). The membranes were washed in Tris buffered saline with Tween 20 (TBS-T) (100 mM Tris–Cl, pH 8.1, 150 mM NaCl, 0.1% Tween-20). Incubation with primary antibody in TBS-T with 1% non-fat milk was performed at 4 °C overnight followed by incubation with horseradish peroxidase conjugated secondary antibody for 1 h and enhanced chemiluminescence detection. The following antibodies and dilutions were used: anti-monocarboxylate transporter 4 (MCT4) (Abcam Inc, Canada; ab74109) 1:1000, anti-glycerol-3-phosphate dehydrogenase-1-like (GPD1L) (ThermoFisher Scientific, USA; PA5-24216) 1:1000, anti-beta Tubulin (Abcam Inc, Canada; ab6046) 1:1000, and anti-hypoxia inducible factor 1 alpha (HIF1a) (Abcam Inc, Canada; ab51608) 1:1000. An anti-rabbit immunoglobulin G (IgG) conjugated to horseradish peroxidase was used as a secondary antibody (New England Biolabs, USA; 7074) at a 1:500 to 1:20,000 dilution. Enhanced chemiluminescence detection on x-ray film was used to detect antibody signal. Equal protein loading was confirmed by re-probing blots for beta-Tubulin. Western blot densitometry values were calculated using FIJI Is Just ImageJ (FIJI).

### Enzyme-linked immunosorbent assays (ELISAs)

Cytokine secretion by primary human astrocytes was measured by ELISA. Forty-eight hours after transfection with 210M or 210S, media was aspirated and fresh media with the appropriate treatment (control or hypoxia and inflammation) was added. Cell supernatants were collected and measured for protein concentration 24 h later. CXCL10 (IP10; BD Biosciences, USA, Cat# 550926), IL-6 (BD Biosciences, USA, Cat# 555220) and insulin-like growth factor-1 (IGF-1) (R&D Systems, USA, Cat# DY291) ELISA kits were used.

### Lactate assay

L-Lactate assay kit (Eton Biosciences, USA, Cat# 1200014002) was used following manufacturer’s instructions on sample supernatants. After the 48 h transfection with 210S or 210M, media was aspirated, wells were washed with 37 °C PBS, and 37 °C serum-free, phenol red-free DMEM F/12 (ThermoFisher, USA 11039-021) fresh media with N1 supplementation (Sigma, Germany, N6530) was added to the cells. Following a 2 h incubation, cell supernatants were collected for measurement of lactate concentration.

### Statistical analyses and data presentation

All data are graphed as the mean ± standard error of the mean (SEM). One-way analysis of variance (ANOVA), two-way ANOVA, or paired *t* tests were performed using Graphpad Prism 9, as noted in the figure legend. A *p*-value < 0.05 was considered significant. For all primary human cell data (except Seahorse data that plots the mean value only), each individual dot represents a sample from an individual human donor. The number of samples per experiment and post-hoc analysis applied is denoted in the figure legends.

## Results

### miRs are differentially expressed in astrocytes from neurologic control tissue compared to those surrounding stroke lesions

**Fig. 1:**
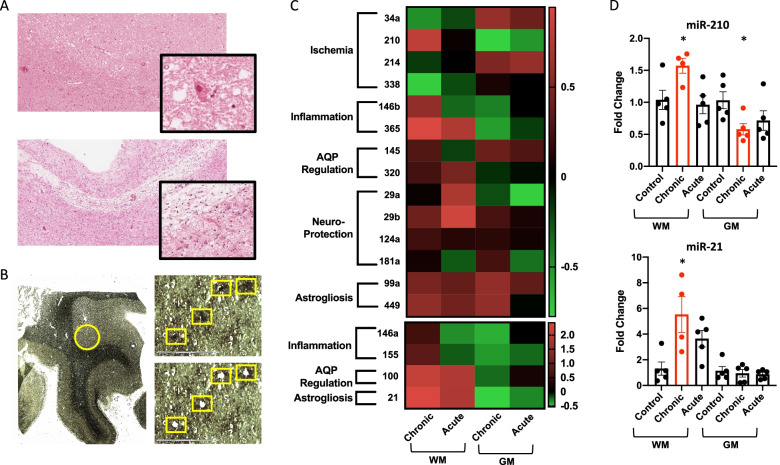
microRNAs are differentially expressed in astrocytes from neurological control tissue versus astrocytes surrounding stroke lesions. **A** Hematoxylin and eosin staining of early acute (upper panel) and late chronic (lower panel) infarcts. Subtle rarefaction/vacuolization of the parenchyma, loss of viable neurons, and acute ischemic, hypereosinophilic neurons (inset) are visible in the acute lesion. Cavitation of the parenchyma with a rim of well-developed astrocytosis (inset) is visible in chronic infarct lesions. **B**–**D** GFAP + astrocytes were captured from white (WM) and gray (GM) matter of unaffected (Neurological Control) brain tissue and around acute or chronic stroke lesions using laser-capture microdissection (LCM). Around 35 cells were pooled from each slide for RT-qPCR assessment of microRNA expression. **B** Bright-field images showing a brain section before and after LCM of astrocytes. **C** Heatmap of microRNA (miR) expression in GFAP + astrocytes. Color code represents the fold change of miR expression in lesioned WM and GM astrocytes compared to respective neurological control astrocytes. miRs are grouped according to their associated function. **D** Histograms of miR-210 and miR-21 expression in GFAP + astrocytes. Data are presented as mean ± SEM of *n* = 5 donors, except for WM C for which *n* = 4. One-way ANOVA was used for significance testing with Dunnett’s multiple comparison test. All miRs with statistically significant differences between neurological control and disease conditions are graphed in red in part D. *p < 0.05

We first aimed to define the miR expression profile of astrocytes around ischemic stroke lesions compared to astrocytes from neurological control tissue. Human brain tissue from both the white matter (WM) and gray matter (GM) of 16 control or stroke patients were obtained. LCM was performed on GFAP + astrocytes from neurological control tissue and from tissue around chronic and acute infarcts (Fig. 1A, B). The age, sex, location of cell capture, type of infarct, and histological characteristics are included for each patient in Table 1. 18 miRs that are known to be expressed by astrocytes and have in silico predicted, or experimentally validated, functions that relate to neuroinflammation or neurodegenerative disease processes were chosen as previously described [49] for RT-qPCR assessment of their expression (Fig. 1C, Additional file 2: Fig. S1, Additional file 1: Table S1). miR-21 and -210 were significantly differentially expressed in astrocytes surrounding infarcts compared to astrocytes from neurological control tissue (Fig. 1C, D). miR-210 was the only miR with significant differential expression around both WM and GM infarcts. Expression of miR-210 was increased ~ 1.6 fold and decreased to ~ 0.6 fold in astrocytes around WM and GM chronic infarcts, respectively (Fig. 1D).

**Table 1:** Histopathological information of human brain autopsies used for laser-capture microdissection of astrocytes

Demographics				Histopathology						Estimated time course	
Sample	Age (years)	Sex	Brain Region	Hemosiderin	Eosinophilic Neurons	Tissue Cavitation	Astrogliosis	Macrophage infiltrate	Swollen capillaries	Estimated interval	Type
1	77	M	WM, GM	Present	Present	Early	Gemistocytic	Confluent	Absent	10 to 30 days	A
2	83	M	WM, GM	Absent	Present	Early	Gemistocytic	Confluent	Absent	2–7 days	A
3	35	M	WM, GM	Absent	Present	Early	Mild	None	Absent	24 h	A
4	53	M	WM, GM	Absent	Present	Early	Mild	Rare	Present	7–10 days	A
5	75	F	WM, GM	Absent	Present	Early	Mild	Rare	Absent	24–48 h	A
6	93	M	WM, GM	Present	Absent	Advanced	Well developed	Rare	Absent	Months to years	C
7	52	F	GM	Present	Absent	Advanced	Well developed	Rare	Absent	Months to years	C
8	63	M	WM, GM	Present	Absent	Advanced	Well developed	Moderate	Absent	Months to years	C
9	80	M	WM, GM	Present	Absent	Advanced	Well developed	Rare	Absent	Months to years	C
10	82	M	WM, GM	Present	Absent	Advanced	Well developed	Rare	Absent	Months to years	C
11	79	M	WM, GM								N
12	50	M	WM, GM								N
13	66	M	WM, GM								N
14	62	M	WM, GM								N
15	30	M	WM, GM								N
16	74	F	WM								N

*M* male, *F* female, *WM* white matter, *GM* gray matter, *A* acute, *C* chronic, *N* neurological control

### miR-210 expression is increased in stressed human astrocytes in vitro and in murine astrocytes in vivo

**Fig. 2:**
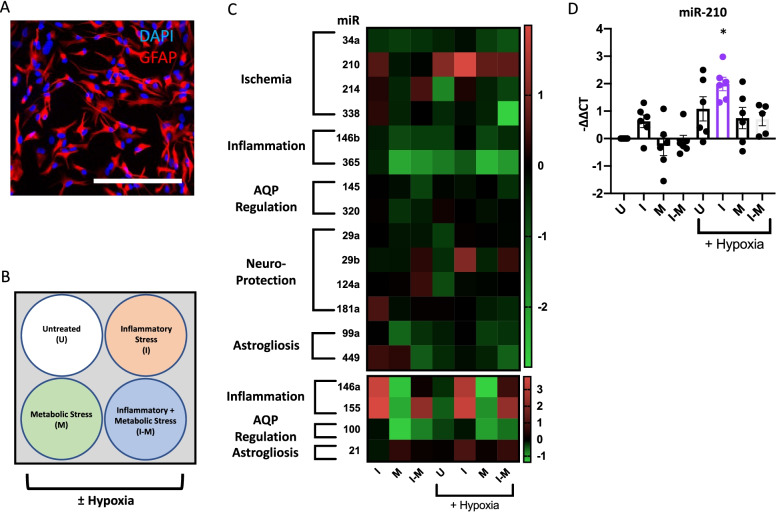
microRNAs are differentially expressed in stressed astrocytes compared to unstressed astrocytes. **A** Primary human fetal astrocytes were stained for expression of Glial Fibrillary Acidic Protein (GFAP) and DAPI to assess purity of human cultures. Scale bar = 500 µm. **B**–**D** Primary human astrocytes were untreated (“U”) or subjected to inflammatory (“I”), metabolic (“M”), and/or hypoxic (“H”) in vitro stress conditions using Interleukin-1 beta (IL1b), glucose-free media, or 1% oxygen, respectively. **C** Heatmap presenting the RT-qPCR assessment of microRNA expression in stress conditions relative to untreated control. Color code represents log_2_(fold change) of miR expression in disease conditions compared to the expression levels in untreated cells. **D** Plot of miR-210 expression levels measured by RT-qPCR. Each dot represents a separate human donor, with 5–6 donors assayed per condition. One-way ANOVA with Sidak’s multiple comparison correction was used for significance testing in image D. *p < 0.05

We hypothesized that the differential miR expression profile observed in situ is an adaptive mechanism in response to cellular stress. We therefore created an in vitro stress paradigm to investigate the modulation of miR expression levels in primary human astrocytes. We first performed immunocytochemistry of human fetal astrocytes and confirmed expression of high levels of the astrocytic marker glial fibrillary acidic protein (GFAP) (Fig. 2A). We further confirmed that the astrocyte cultures were negative for any microglia/oligodendrocyte markers at the mRNA level (Additional file 3: Fig. S2A), and we quantified the GFAP staining of astrocytes compared to microglia/oligodendrocyte markers (Additional file 3: Fig. S2B). Astrocytes were subsequently exposed to inflammatory, metabolic, and hypoxic stress, as well as combination of these conditions, using IL1b treatment, glucose-free medium, and a 1% O_2_ chamber, respectively (Fig. 2B). After 24 h, expression of canonical stress response genes were measured and found to be upregulated in cells subjected to inflammatory stress (*CXCL10* [30]), metabolic stress (hemeoxygenase 1 (*HMOX1)* [6, 28]), and hypoxic stress (*MCT4* [52]) (Additional file 3: Fig. S2C). Increased HIF-1α protein expression is regarded as the best indicator of hypoxic stress, but it could not be readily detected in astrocytes using immunoblotting after hypoxic insult (data not shown). To ensure that hypoxia was successfully induced in our conditions, HeLa cells cultured in parallel were cultured in the hypoxia chamber and were shown to increase expression of HIF-1α after 2-, 4-, and 24 h (Additional file 3: Fig. S2D). Following establishment of the in vitro stress paradigm, the expression levels of the 18 miRs with functional relevance to astrocytes [49] were analyzed by RT-qPCR (Fig. 2C, Additional file 4: Fig. S3). miR-210 was significantly upregulated in astrocytes exposed to a combination of hypoxia and inflammation (Fig. 2D). Other miRs followed previously identified patterns, such as miR-155, which was increased in the inflammatory conditions [34]. The expression of miR-210 in situ, the modulation of miR-210 in astrocytes exposed to hypoxia/inflammation, and the previously documented upregulation of miR-210 in WM astrocytes around MS lesions [48] highlighted miR-210 as a miR of significant interest. To investigate the modulation of miR-210 expression in vivo, microRNAscope (miRscope) was used to measure miR-210 in the middle cerebral artery occlusion (MCAO) murine stroke model. We obtained mouse tissue 14-days after induction of MCAO and used miRscope to image and quantify miR-210 in cells directly around MCAO infarcts compared to cells in the contralateral region of the brain (Additional file 3: Fig. S2E). We found that a higher proportion of cells were positive for miR-210 around the MCAO infarct lesions than in the contralateral tissue (Additional file 3: Fig. S2F). Altogether, this data further supported the investigation of miR-210 in vitro.

### Primary human astrocytes can be transfected with miR-210-mimic

**Fig. 3:**
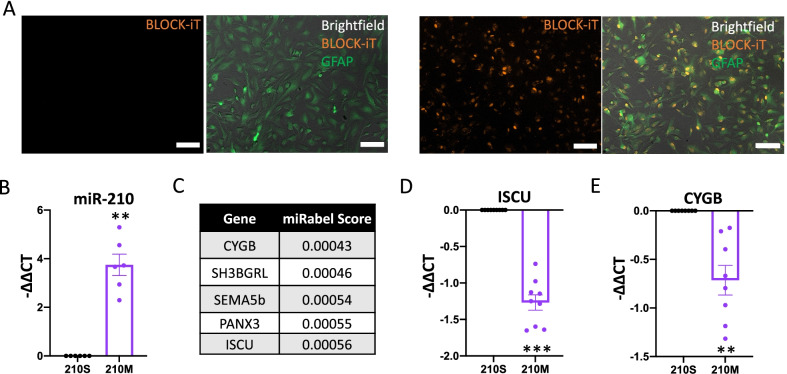
Primary human astrocytes downregulate known targets of miR-210 following transfection with miR-210-Mimic. **A** Primary human astrocytes were transfected with AlexaFluor BLOCK-iT as a positive control of transfection. Representative images of non-transfected (left) and transfected (right) cells immunostained for GFAP and overlayed with brightfield. Scale bar = 100 µm. **B** Primary human astrocytes were transfected with miR-210-Mimic (210M) or miR-210-Seed-mutant (210S) as a control. The expression of miR-210 in 210M-transfected relative to 210S-transfected cells was assessed by RT-qPCR, *n* = 6. **C** MiRabel analysis of miR-210 shows the five most likely gene-targets of miR-210 based on sequence-specific predicted targeting, with lower MiRabel scores signifying a higher likelihood of being targeted by miR-210. **D** RT-qPCR assessment of microRNA-210 targets Iron Sulfur Cluster Protein (*ISCU*, *n* = 9) and **E** Cytoglobin (*CYGB*, *n* = 8) in 210M-transfected relative to 210S-transfected cells. All data is graphed as mean ± SEM. Each dot represents a separate human sample. Paired *t* tests were performed for tests of significance. *p < 0.05; **p < 0.01; ***p < 0.001

To elucidate the function of miR-210 in astrocytes, we used a miR mimic system. The miR mimic (210M) has the same sequence as endogenous miR-210 and therefore acts as an in vitro overexpression model. As a negative control, we generated a miR-210 construct with a transposed seed sequence between the miR-210-3p and miR-210-5p, hereafter referred to as 210S [17]. To confirm that lipid-mediated transfection of primary human astrocytes is possible, we first verified efficient transfection of the cells with Alexa Fluor 555 BLOCK-iT, a fluorescently labelled short nucleotide sequence. GFAP-positive astrocytes showed an increase in Alexa Fluor 555 fluorescence signal after 48 h compared to non-transfected cells (Fig. 3A). Cells were simultaneously imaged using bright field microscopy to display all cells in the field. Brightfield was chosen over DAPI, because the DAPI signal overpowered the Block-IT signal in many of the cells. Next, intracellular astrocytic miR-210 expression levels were measured by RT-qPCR after transfection with 210M or 210S. Primary human astrocytes transfected with 210M had significantly higher levels of miR-210 relative to cells transfected with the control 210S (Fig. 3B). To confirm the functionality of 210M, we measured the expression levels of known downstream targets of miR-210. The MiRabel bioinformatic tool, which uses predictive algorithms to identify likely gene targets of a given miR [47], was used to identify miR-210 targets (Fig. 3C). From this target list, we measured the expression of cytoglobin (*CYGB)* and iron sulfur cluster protein (*ISCU)*, two experimentally validated targets of miR-210 [7, 10]. We found that mRNA levels of both *CYGB* and *ISCU* were significantly downregulated in astrocytes transfected with 210M relative to 210S (Fig. 3D, E). Altogether, these data demonstrate that primary human astrocytes are capable of being transfected with miR mimics using lipid-mediated delivery, and that transfecting astrocytes with 210M results in the downregulation of miR-210 target genes, suggesting that 210M is functionally active within these cells.

### 210M promotes glycolysis and lactate export in primary human astrocytes

**Fig. 4:**
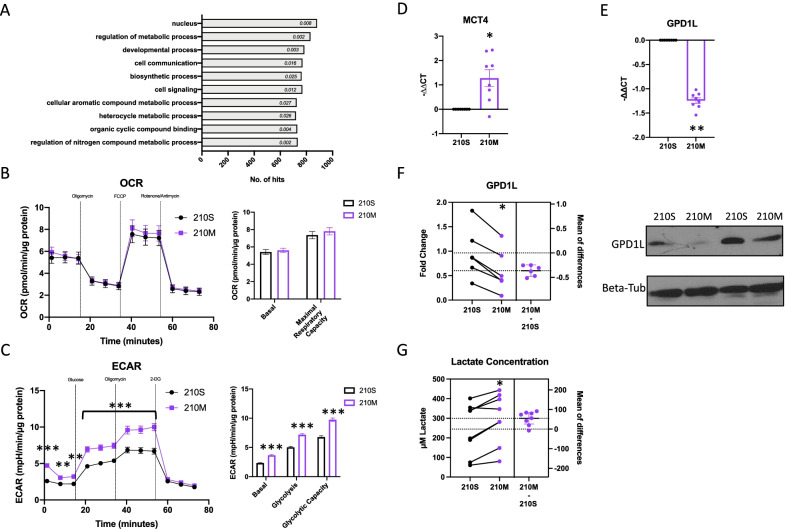
210M induces glycolysis and lactate export of primary human astrocytes. **A** miRPathDB was used to predict pathways targeted by miR-210. The pathways with the most hits are listed in descending order, and the *p*-value of each pathway is included inside the respective bar. **B** Oxygen Consumption Rate (OCR) was measured in primary human astrocytes 48 h after transfection with 210S or 210M. The basal oxygen consumption (before oligomycin) and maximal respiratory capacity (between FCCP and Rotenone/Antimycin A) are presented in the histogram. *n* = 3 with 10 technical replicates for OCR experiments. **C** Extracellular Acidification Rate (ECAR) was measured in primary human astrocytes 48 h after transfection with 210S or 210M. The basal (before glucose), normal glycolysis (between glucose and oligomycin) and glycolytic capacity (between oligomycin and 2-DG addition) are presented in the histogram. *n* = 4 with 20 technical replicates for ECAR experiments. **D** RT-qPCR assessment of *MCT4* in 210M-transfected cells relative to 210S-transfected cells 48 h after transfection. Mean ± SEM of 8 donors. **E** RT-qPCR assessment of *GPD1L* in 210M-transfected cells relative to 210S-transfected cells 48 h after transfection. Mean ± SEM of 8 donors. **F** Immunoblotted bands of GPD1L and Beta-tubulin (Beta-Tub) proteins and their quantification in 210M-transfected relative to 210S-transfected cells 48 h after transfection. Mean ± SEM of 6 donors. **G** Primary human astrocytes were transfected with 210S or 210M. After 48 h, cells were washed, and the concentration of lactate was measured in media that was incubated with astrocytes for 2 h. Mean ± SEM of 8 donors. Statistical comparisons were made by two-way ANOVA with Sidak’s correction (**B** and **C**) or by a paired *t*-test (**D**–**H**). *p < 0.05; **p < 0.01; ***p < 0.001

The bioinformatic software miRPathDB [24] was used to predict pathways that could be regulated by miR-210. For miR-210-3p, the functional pathway with the 2nd most hits was the “regulation of metabolic process”, and many of the other top pathways regulated by miR-210 were also related to metabolism (cellular aromatic compound, organic cyclic, and nitrogen compound metabolic processes) (Fig. 4A). A Seahorse XF analyzer was therefore used to investigate the effect of miR-210 expression on primary human astrocyte metabolism. The oxygen consumption rate (OCR) and extracellular acidification rate (ECAR), which are respective proxies for oxidative phosphorylation and glycolytic activity, were assessed. 24 h post-transfection, there was no change in either the OCR or ECAR in cells transfected with 210M compared to those transfected with 210S (Additional file 5: Fig. S4A, B). 48 h post-transfection, there remained no significant change in OCR values between astrocytes transfected with 210M or 210S (Fig. 4B). Conversely, ECAR values of basal glycolysis, normal glycolysis, and glycolytic capacity were significantly increased in astrocytes transfected with 210M (Fig. 4C). The timepoint of 48 h was therefore chosen to investigate the effect of 210M transfection on lactate export, a metabolite produced through glycolysis that has previously been shown to be critical for neuronal survival in stressful conditions [38]. First, we measured the expression of monocarboxylate transporter 4 [MCT4, also known as solute carrier 16A3 (SLC16A3)], which is a monocarboxylate transport protein that exports lactate from astrocytes, expression of which has previously been shown to be critical for neuronal survival under states of oxygen and glucose deprivation [13]. In astrocytes transfected with 210M, a ~ 2.2-fold increase in the expression of *MCT4* mRNA compared to those transfected with 210S was observed (Fig. 4D). MCT4 protein expression in astrocytes transfected with 210M was also found to be increased by Western blot compared to astrocytes transfected with 210S, though this difference did not reach statistical significance (Additional file 5: Fig. 4C). In addition, both mRNA and protein levels of GPD1L, which was previously shown to inhibit lactate production when overexpressed [10], were decreased in astrocytes transfected with 210M compared to cells transfected with 210S (Fig. 4E, F). We also found that the mRNA expression of lactate dehydrogenase A* (LDHA),* which is involved in the production of lactate from pyruvate*,* was upregulated in astrocytes transfected with 210M (Additional file 5: Fig. 4D). Finally, to understand if the combined effect of increased MCT4 expression and decreased GPD1L expression had an effect on the release of astrocytic lactate into the extracellular space, we evaluated lactate accumulation in the supernatant of astrocytes over a 2 h period, 48 h after transfection with 210M or 210S. We found that astrocytes transfected with 210M exported ~ 1.3-fold more lactate than the 210S transfected cells (Fig. 4G). Overall, 210M transfection promoted glycolysis in primary human astrocytes, resulting in increased lactate export.

### 210M decreases inflammatory cytokine production of primary human astrocytes

**Fig. 5:**
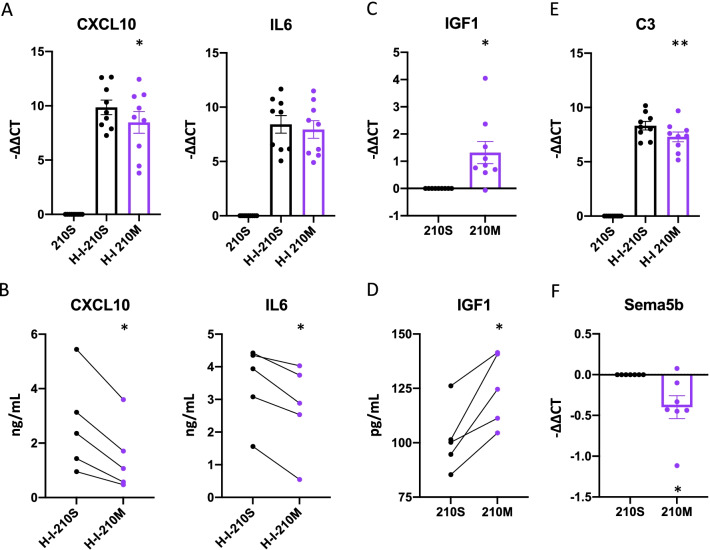
210M transfected astrocytes exhibit an anti-inflammatory and neuroprotective phenotype. Primary human astrocytes were transfected with 210S or 210M, with or without prior exposure to hypoxic and inflammatory stress (“H-I”) using 1% oxygen and interleukin 1-beta, respectively. **A**, **C** RT-qPCR assessment of *CXCL10, IL6, IGF1* gene expression. **B**, **D** CXCL10, IL6, and IGF1 protein levels in cell supernatant measured by ELISA. **E**, **F** RT-qPCR assessment of *C3* and *Sema5b* gene expression. All data are presented as mean ± SEM of *n* = 9 (**A**, **C** and **E**), 5 (**B** and **D**) or 7 (**F**) donors. Statistical comparisons between groups were made by a one-way ANOVA (A and E) or by a paired *t*-test (**B**–**D**, **F**). *p < 0.05; **p < 0.01

miR-210-mediated increased lactate production by astrocytes may indicate the establishment of a neuroprotective astrocytic phenotype [4]. To further investigate this possibility, the effect of miR-210 overexpression on pro-inflammatory cytokine (IL-6 and CXCL10) and protective growth factor (IGF-1) [54] production in primary human astrocytes was evaluated. As the expression of pro-inflammatory cytokines are low in astrocytes under basal conditions, cells were incubated with 210M or 210S for 48 h and then stressed for 24 h in combined hypoxic and inflammatory condition. Under this condition, decreased levels of CXCL10 mRNA and secreted protein (Fig. 5A, B) were observed in 210M- compared to 210S-transfected cells. The expression of *IL-6* mRNA also trended downwards with 210M transfection, while the cytokine level of IL-6 as measured by ELISA was significantly lower following 210M transfection compared to 210S transfection (Fig. 5A, B). In addition to decreasing pro-inflammatory cytokine expression, astrocytes transfected with 210M were characterized by increased mRNA and secreted protein levels of the growth factor IGF-1 (Fig. 5C, D). To further explore how increased miR-210 expression alters human astrocyte phenotype under stressed condition, we measured gene expression levels of complement 3 (*C3*), which has been found to be a driver of neuroinflammation associated with neuronal loss and the suppression of axonal growth [43]. We found that *C3* mRNA levels were lower in cells transfected with 210M as compared to those transfected with 210S (Fig. 5E). We also found that *Sema5b*, which is a repressive cue for axonal growth [40], was decreased at the mRNA level with 210M transfection (Fig. 5F). Protein expression levels of C3 and Sema5b were not measured. We investigated the mRNA expression levels of *Rela, Nfkb, Nos2, Nfe2l2,* and *S100A10*, which have all been identified as either promoters or repressors of astrocytic inflammatory pathways [29, 58]. The expression of these genes was unchanged by 210M transfection either with or without inflammatory stimulus (Additional file 6: Fig. S5). Overall, these findings confirm that miR-210 has anti-inflammatory effects in primary human astrocytes undergoing stroke-like stress, and that 210M may promote increased interaction between astrocytes and neurons through the decreased expression of *C3* and *Sema5b.*

## Discussion

Here we used laser-capture microdissection (LCM) to capture astrocytes and identify the astrocytic miR expression profile from around human stroke lesions in both WM and GM. In our data, miR-210 was upregulated in WM astrocytes but was conversely downregulated in GM astrocytes around chronic stroke lesions. Neuronal extensions in the WM rely more heavily on astrocyte-derived metabolic products for energy due to their limited axonal surfaces when compared to the large cellular surface area of cells in the GM [4]. This may explain the differential regulation of miR-210 in WM and GM astrocytes. In the weeks/months following an ischemic stroke, the increased energy demand required to promote cell survival and repair damaged neuronal networks may drive a reliance on astrocyte-derived lactate for energy in WM tissue. Additional studies are needed to fully understand the differential expression of miR-210 in WM and GM astrocytes.

In addition to observing significant changes in miR-210 expression in astrocytes around stroke lesions compared to the neurological control astrocytes, our in-situ analysis uncovered other differentially expressed astrocytic miRs that we have not investigated further in the current study. For instance, miR-21 was significantly upregulated in astrocytes around WM chronic infarcts, suggesting that it may play a role in the astrocytic response to disease in the WM. Likewise, miR-365 was upregulated in astrocytes around WM chronic and acute infarcts, however this finding did not reach statistical significance. Similar to miR-210, miR- 29a and -29b, two miRs that have been associated with neuroprotection [16, 25], were non-significantly upregulated in astrocytes around WM chronic infarcts, suggesting that high miR-210 levels may be indicative of an overall neuroprotective astrocyte signature.

The laser-capture data also allowed us to study the differences in miR expression between astrocytes surrounding chronic and acute lesions. We believe that the differential expression profile of miRs in chronic and acute lesions is a result of astrocytes responding to the evolution of the local cellular stress environment over time. During the acute phase of an ischemic stroke, which includes the initial hours-to-days of damage, extensive cell death results from the onset of hypoxia, reduced glucose perfusion, increased levels of inflammatory cytokines, and the release of danger associated molecular patterns from apoptotic or necrotic cells. Chronic stroke involves longer term evolution of changes to the cellular environment driven by gliosis and tissue remodeling [5]. These two timepoints of disease (chronic and acute), therefore, contain their own unique cellular stress responses that may be responsible for the differential miR expression profile observed between chronic and acute stroke lesions. In fact, astrocytes in chronic stroke lesions may not just be responding to present stresses but may have been influenced by previous changes in their miR and protein expression profile. The concept of ischemic preconditioning, in which cells become more resistant to ischemic stresses after surviving an initial insult [26, 57], suggests a mechanistic framework to explain why astrocytes around chronic stroke lesions express increased levels of miR-210. We propose that chronic stroke is a primary indication for which neuroprotective therapeutic strategies may be successful. While identifying treatments for acute ischemic stroke to supplement existing thrombolytic therapies remains a public health imperative, disease modifying therapies for the persistent neurologic deficits that follow ischemic infarcts remain largely unexplored [3].

To investigate the functionality of miRs, we created an in vitro system that allowed for manipulation of miR expression levels. Inflammatory, metabolic, and hypoxic stresses are known to be involved in stroke pathogenesis and were thus the focus of our in vitro stress paradigm [2]. This combination of stresses is not meant to perfectly recapitulate the complex stroke environment, as the crosstalk between cells alone is too complex to recreate using this reductionist in vitro approach. Instead, this system provides a method to investigate specific miRs of interest identified in-situ. For instance, miR-210 was robustly increased under combined hypoxic and inflammatory stress condition. While this condition does not perfectly recapitulate the chronic stroke microenvironment, it combined known disease-relevant stresses and more importantly it established an in vitro miR-210 signature in astrocytes that mirrored what we observed in astrocytes captured directly from the human stroke brain. The overexpression of miR-210 in astrocytes increased glycolysis and anti-inflammatory cytokine production, but it also decreased the expression of genes related to neurotoxicity that were upregulated in the hypoxic and inflammatory stress condition (Figs. 4 and 5).

The astrocyte-neuron lactate shuttle hypothesis (ANLSH) states that astrocyte-derived lactate is shuttled to neurons as an additional energy source, and it is shuttled both under homeostatic and disease conditions [14]. Lactate can be converted to pyruvate by the actions of lactate dehydrogenase in neurons (an anaerobic process) and this pyruvate is then used in the citric acid cycle and oxidative phosphorylation processes. While the ability of astrocyte-derived lactate to protect neurons during ischemic stress remains under investigation, this metabolite has been extensively shown to confer an overall protective effect to neurons when energetic demands are high [4]. Blockade of the lactate exporter MCT4, which we observed to be increased in astrocytes transfected with 210M, has previously been shown to lead to decreased neuronal survival under oxygen and glucose deprivation conditions, providing evidence that astrocyte-derived lactate is crucial in supporting neuronal survival under stressful conditions [13, 19]. Increased lactate export resulting from increased astrocytic miR-210 in white matter tissue may also relate to ischemic preconditioning mentioned earlier in the Discussion section. Conversely, other studies have shown that increased astrocyte-derived lactate can lead to acidosis-mediated-neurotoxicity [59]. The subtle increase in lactate export observed in this study following 210M transfection (~ 1.3 fold) is unlikely to cause neurotoxicity while still providing an additional energy source for surrounding cells. Although the mechanism of increased lactate export was only partially elucidated in this study (through decreased GPD1L, decreased ISCU, and increased gene expression), previous studies have found that miR-210 activates HIF-1a, which may be responsible for driving broad cellular changes to increase glycolysis and thus lactate export.

Astrocytes are important contributors to the inflammatory response in the brain, primarily through the expression and secretion of inflammatory cytokines. Post-thrombolytic inflammation is one of the primary drivers of cell stress and death in stroke, and inflammation is also a central component of other neurodegenerative diseases like progressive MS [39]. Having observed a miR-210-mediated increase in glycolysis (Fig. 4), we hypothesized that astrocytes transfected with 210M would also increase production of pro-inflammatory cytokines, as increased glycolysis often simultaneously increases activity of inflammatory pathways [41]. Interestingly we observed decreased expression of the pro-inflammatory cytokines IL-6 and CXCL10, and increased expression of the protective mediator IGF-1 (Fig. 5). This supports previous evidence that miR-210 targets proteins involved in the nuclear factor kappa-light-chain-enhancer of activated B cells (NF-kB) pathway to inhibit inflammatory cascades [46], though we did not investigate NF-kB protein levels in this study. We also investigated the expression of previously defined “A1” or “A2” astrocytic markers that are thought to define neurotoxic or neuroprotective astrocyte phenotypes, respectively [29]. Though the terms A1 and A2 are on longer used [12], many of the genes identified to be either toxic or protective (A1 or A2 respectively) were of interest in our study. However, we saw no changes in these markers (Additional file 6: Fig. S5), with the exception of decreased *C3* expression when astrocytes were transfected with 210M (Fig. 5). *C3* is defined as an ‘A1 marker’ but is more broadly associated with inflammatory cascades and which is implicated in mediating cytotoxicity. Additionally, expression of *Sema5b*, a molecule that represses axon guidance and outgrowth [40], was also inhibited following 210M transfection. Based on this data, we can speculate that decreased expression of Sema5b by astrocytes with a neuroprotective phenotype (through increased lactate export and decreased inflammatory cytokine production) may serve to attract neurons in a post-damage environment. Previous studies have also shown that primary human fetal astrocytes produce TNF in response to IL-1b treatment [23, 27], but we did not measure it in this study because other pro-inflammatory cytokines are produced at higher levels than TNF, and because more TNF is produced by microglia than by astrocytes.

Finally, a potential limitation of this study is that our in-situ investigation of miRs in astrocytes around stroke lesions was limited to a pre-defined list of miRs. In our initial screening of the astrocytic miR profile, we selected 18 miRs based on previous publications that have defined these miRs to be involved in the astrocytic response to disease. Obtaining the necessary tissue to perform sequencing experiments and to subsequently confirm those findings at the PCR level would have not been feasible with the tissue available. Additionally, the small sample size for the in-situ experiment likely restricted the differential expression of certain miRs from reaching statistical significance. Despite this, our data shows trends of increased or decreased miR expression around stroke lesions and should provide a basis for further study of astrocytic miRs and their functions. Importantly, although we took care to capture only GFAP + DAB-stained cells that displayed morphologic features consistent with astrocytes (as confirmed by an experienced neuropathologist), neither mRNA nor protein could be assessed in the captured cells to confirm absolute purity. Additionally, access to primary human astrocytes limited the breadth of in vitro experiments that could be performed. The use of primary human fetal astrocytes, though likely more applicable to adult cells than immortalized or murine astrocytes, retain differences compared to adult cells, and so conclusions made from the use of fetal cells must be taken with this point in mind. For the in vitro experiments, we focused on using 210M as an overexpression model of miR-210; however, as alternative sources of human astrocytes including induced pluripotent stem cell-derived astrocytes become more widely available, additional experiments using miR-210 inhibitors and miR-210 knock-out approaches could be used to explore this biology further, which is necessary to fully understand how miR-210 affects astrocytes. Increased access to human astrocytes will also allow for better optimization of miR-mimic and inhibitor concentration. In this study, we used a high concentration of miR-210 mimic (50 nM), which resulted in an increased expression of miR-210 in cells that is greater than that which is physiologically relevant. In past studies, use of high-dose miR-mimic (100 nM) was shown to cause off target effects [22] and, therefore, this is an important consideration for future studies. This would also enable us to more thoroughly investigate the genes impacted by miR-210 using sequencing or microarray experiments, which were not feasible in this study due to limited cell numbers. Notwithstanding these limitations, this study suggests that miR-210 is an important regulator of astrocytic function in ischemic tissue.

## Conclusion

In summary, we found that human astrocytes around WM chronic stroke lesions have increased miR-210 expression compared to astrocytes in neurological control tissue. Increased miR-210 expression boosted astrocytic glycolytic activity, enhanced lactate production, and increased expression of anti-inflammatory molecules while inhibiting the expression of pro-inflammatory mediators. Overall, we propose a functional role for miR-210 in promoting beneficial functions of astrocytes that may be critical in promoting neuronal survival after stroke. While further research is required, this study expands our knowledge of disease-associated astrocyte signatures and identifies miR-210 as a potentially modifiable mediator to promote a protective astrocytic phenotype.

## Supplementary Information

**Additional file 1: Table S1.** Mean cycle threshold values of microRNAs from white matter and gray matter astrocytes.

**Additional file 2: Figure S1.** MicroRNA expression profile of astrocytes from neurological control tissue and around ischemic stroke lesions. GFAP + astrocytes were captured from white (WM) and gray (GM) matter of neurological control (“N”) brain tissue or around acute (“A”) or chronic (“C”) stroke lesions using laser-capture microdissection (LCM), and the expression levels of the listed miRs were then measured by RT-qPCR. Expression levels of white matter astrocytes are relative to the WM N condition, whereas expression levels of gray matter astrocytes are relative to the GM N condition. No graphs listed reached significance using one-way ANOVAs with Dunnett’s multiple comparison test. *n* = 4–5.

**Additional file 3: Figure S2.** Primary human fetal astrocytes respond to in vitro stresses, and mice upregulate miR-210 around ischemic lesions. (A) Primary human astrocytes, oligodendrocytes, and microglia were separately cultured and analyzed for expression of canonically expressed genes of glial fibrillary acidic protein (*GFAP*), myelin basic protein (*MBP*) and ionized calcium binding adaptor molecule 1 (*IBA1*) by RT-qPCR. *n* = 3–4. (B) Primary human fetal astrocytes were immunostained with anti-GFAP, anti-O4, and anti-PU.1 antibodies. A CX7 automated microscope was used to quantify the percent positive astrocytes for each marker, *n* = 4. (C) Primary human astrocytes were treated in inflammatory (“I”) metabolic (“M”) or hypoxic (“H”) stress conditions induced by IL1b, glucose-free media, or a 1% oxygen chamber for 24 h compared to untreated control. RT-qPCR assessment of *CXCL10*, *HMOX1*, and *MCT4* expression was measured to confirm the astrocytic response to inflammatory, metabolic, and hypoxic stress conditions, respectively, *n* = 4–6. (D) HeLa cells were left in normoxic (“N”) conditions for 48 h or were treated in 1% O2 (hypoxia, H) for the noted time. After 2–48 h, the cells were removed and were immediately lysed with RIPA buffer for subsequent Western blot. *n* = 3. (E) Graphical representation of the area investigated for miR-210 expression using miRscope. miR-210 was quantified in the highlighted region around the infarct site (left), and in the respectively highlighted region on the contralateral side of the brain (right). 300–500 cells were counted per brain section. (F) Representative images and quantification of microRNAscope (miRscope) of miR-210 around MCAO lesions (labeled as infarct) or on the contralateral side of the brain (labeled as control). Scale bar = 20 µm. Quantification was performed by manual counting of the number of nuclei positive for one or more red puncta. Red dots show miR-210 expression, and DAPI (blue) was used as a counterstain. Each dot in image F represents a separate tissue section all derived from one mouse, resulting in five sections imaged per brain region.

**Additional file 4: Figure S3.** microRNAs are differentially expressed in primary human astrocytes undergoing different stresses. (A) Primary human astrocytes, oligodendrocytes, and microglia were separately cultured and analyzed for expression of canonically expressed genes of glial fibrillary acidic protein (*GFAP*), myelin basic protein (*MBP*) and ionized calcium binding adaptor molecule 1 (*IBA1*) by RT-qPCR. *n* = 3–4. (B) Primary human fetal astrocytes were immunostained with anti-GFAP, anti-O4, and anti-PU.1 antibodies. A CX7 automated microscope was used to quantify the percent positive astrocytes for each marker, *n* = 4. (C) Primary human astrocytes were treated in inflammatory (“I”) metabolic (“M”) or hypoxic (“H”) stress conditions induced by IL1b, glucose-free media, or a 1% oxygen chamber for 24 h compared to untreated control. RT-qPCR assessment of *CXCL10*, *HMOX1*, and *MCT4* expression was measured to confirm the astrocytic response to inflammatory, metabolic, and hypoxic stress conditions, respectively, *n* = 4–6. (D) HeLa cells were left in normoxic (“N”) conditions for 48 h or were treated in 1% O_2_ (hypoxia, H) for the noted time period. After 2–48 h, the cells were removed and were immediately lysed with RIPA buffer for subsequent Western blot. *n* = 3.

**Additional file 5: Figure S4.** Astrocytic Metabolism and MCT4 Protein Expression is unchanged after 24- and 48-h 210M transfection, respectively. (A) Oxygen Consumption Rate (OCR) was measured in primary human astrocytes 24-h after transfection of 210S or 210M. *n* = 4 with 10 technical replicates for OCR experiments. (B) Extracellular Acidification Rate (ECAR) was measured in primary human astrocytes 24-h after transfection of 210S or 210M. *n* = 4 with 20 technical replicates for ECAR experiments. (C) Protein expression of MCT4 and Beta Tubulin (Beta-Tub) were measured by western blot in six separate human samples. Data is graphed as the fold change of MCT4 expression normalized to Beta-Tubulin expression and relative to the expression of 210S. For (C), each dot represents a separate human sample. (D) RT-qPCR assessment of *LDHA* in 210M-transfected cells relative to 210S-transfected cells 48 h after transfection. Mean ± SEM of 6 donors. Two-way ANOVA with Sidak’s correction was performed for Seahorse experiments, whereas t-test was used for MCT4 and *LDHA* expression. *p < 0.05.

**Additional file 6: Figure S5.** 210M does not affect astrocytic expression of select pro- or anti-inflammatory genes. Select genes that have previously been identified to promote or antagonize inflammation in astrocytes were measured by RT-qPCR in primary human fetal astrocytes. Cells were transfected with 210S or 210M and left otherwise untreated or treated with hypoxia (H) and inflammation (I) using 1% oxygen and IL1b, respectively. *n* = 3–4, data is graphed as mean ± SEM and was analyzed using one-way ANOVA with Sidak’s multiple comparison test.

## Data Availability

The datasets used and analysed during the current study are available from the corresponding author upon reasonable request.

## Abbreviations

miR: MicroRNA
RT-qPCR: Reverse transcription-quantitative polymerase chain reaction
ELISA: Enzyme-linked immunosorbent assay
210M: MiR-210-mimic
C3: Complement 3
Sema5b: Semaphorin 5b
CNS: Central nervous system
BBB: Blood brain barrier
LCM: Laser-capture microdissection
WM: White matter
GM: Gray matter
MS: Multiple sclerosis
FFPE: Formalin-fixed paraffin-embedded
GFAP: Glial fibrillary acidic protein
HRP: Horse radish peroxidase
DNA: Deoxyribonucleic acid
RNA: Ribonucleic acid
cDNA: Complementary deoxyribonucleic acid
CT: Cycle threshold
mRNA: Messenger ribonucleic acid
GAPDH: Glyceraldehyde 3-phosphate dehydrogenase
PBS: Phosphate buffered saline
PS: Penicillin–streptomycin
FBS: Fetal bovine serum
IL1b: Interleukin-1 beta
MEM: Minimum essential medium
PDGF: Platelet-derived growth factor
bFGF: Basic fibroblast growth factor
T3: Triiodothyronine
210S: MiR-210-Seed
IDT: Integrated DNA Technologies
OCR: Oxygen consumption rate
ECAR: Extracellular acidification rate
FCCP: Carbonyl cyanide-4 (trifluoromethoxy) phenylhydrazone
MCT4: Monocarboxylate transporter 4
GPD1L: Glycerol-3-phosphate dehydrogenase-1-like
HIF1a: Hypoxia inducible factor 1 alpha
IgG: Immunoglobulin G
FIJI: FIJI Is Just ImageJ
IL-6: Interleukin-6
CXCL10: C-X-C chemokine ligand 10
IGF-1: Insulin-like growth factor 1
ANOVA: Analysis of variance
HMOX1: Hemeoxygenase 1
CYGB: Cytoglobin
ISCU: Iron sulfur cluster protein
SLC16A3: Solute carrier 16A3
ANLSH: Astrocyte-neuron lactate shuttle hypothesis
