# Supplementary material for: MicroRNA-210 regulates the metabolic and inflammatory status of primary human astrocytes
Source: J Neuroinflammation. 2022 Jan 6;19:10. doi: 10.1186/s12974-021-02373-y (PMC8740343; doi:10.1186/s12974-021-02373-y)
Supplement: Supplementary file 1 — Additional file 1: Table S1. Mean cycle threshold values of microRNAs from white matter and gray matter astrocytes. [file 12974_2021_2373_MOESM1_ESM.pdf]

miR-	WM CT Value Mean	GM CT Value Mean
34a	28.72	28.50
210	32.85	31.70
214	35.82	35.99
338	25.16	24.99
146b	33.49	32.42
365	30.73	30.35
145	28.83	28.31
320	31.50	30.83
29a	32.50	32.39
29b	32.57	32.73
124a	31.33	31.33
181a	31.43	30.97
99a	29.10	28.35
449	27.13	27.47
146a	30.24	29.59
155	33.49	33.70
100	29.32	27.34
21	32.41	31.88

Legend	
**miR**	microRNA
**CT**	Cycle Threshold
WM	White Matter
GM	Gray Matter
