# Supplementary figures and images for: MicroRNA-210 regulates the metabolic and inflammatory status of primary human astrocytes
Source: J Neuroinflammation. 2022 Jan 6;19:10. doi: 10.1186/s12974-021-02373-y (PMC8740343; doi:10.1186/s12974-021-02373-y)

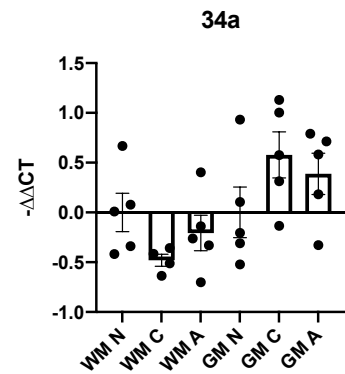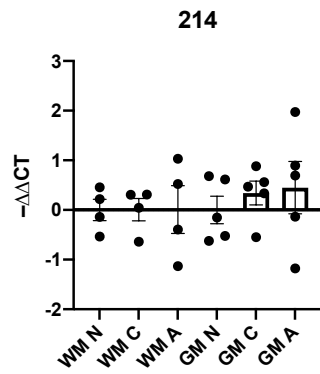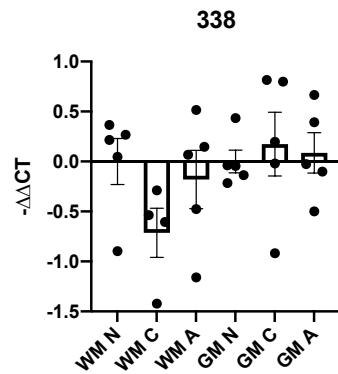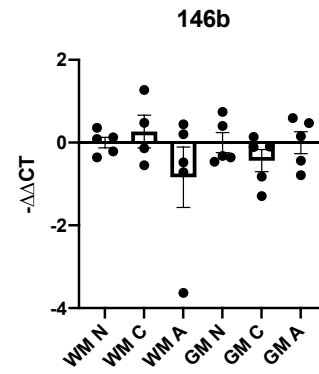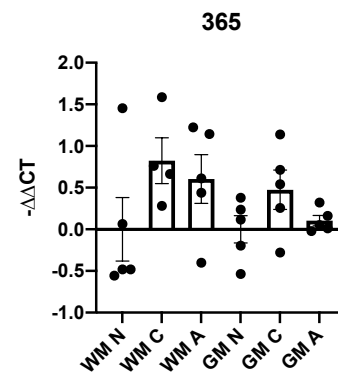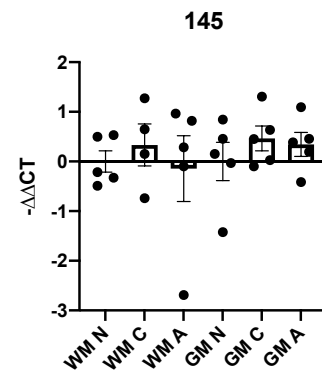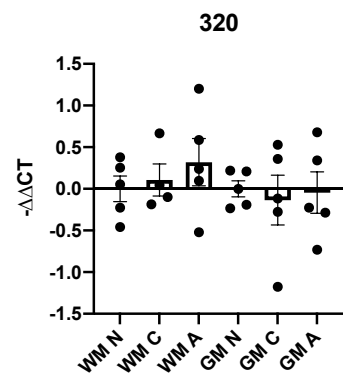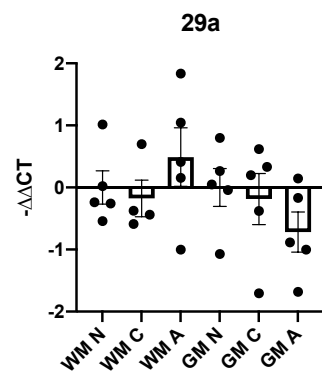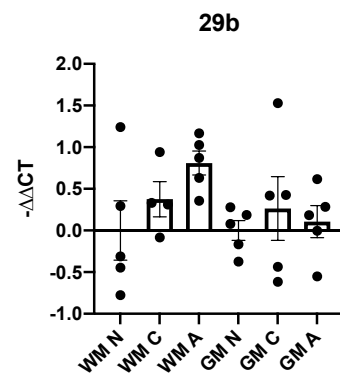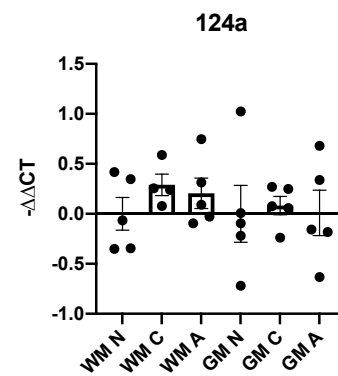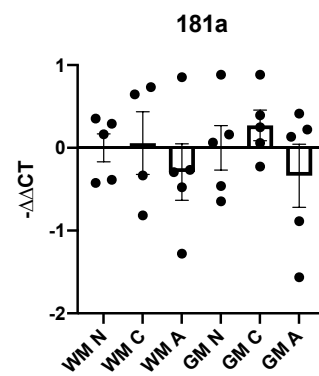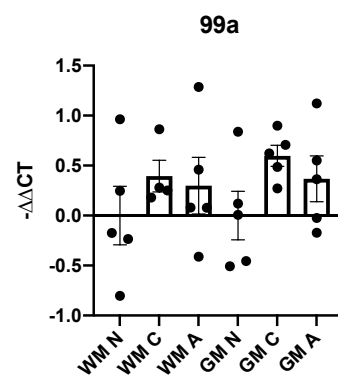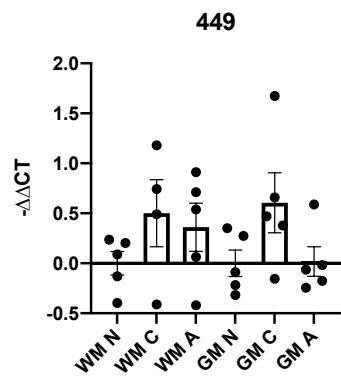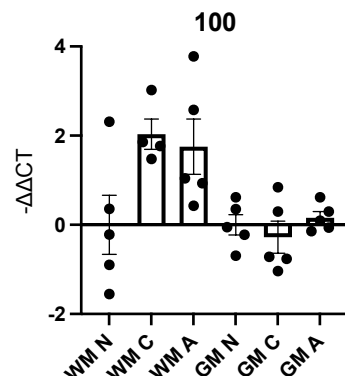

Supplement: Supplementary file 2 — Additional file 2: Figure S1. MicroRNA expression profile of astrocytes from neurological control tissue and around ischemic stroke lesions. GFAP + astrocytes were captured from white (WM) and gray (GM) matter of neurological control (“N”) brain tissue or around acute (“A”) or chronic (“C”) stroke lesions using laser-capture microdissection (LCM), and the expression levels of the listed miRs were then measured by RT-qPCR. Expression levels of white matter astrocytes are relative to the WM N condition, whereas expression levels of gray matter astrocytes are relative to the GM N condition. No graphs listed reached significance using one-way ANOVAs with Dunnett’s multiple comparison test. n = 4–5. [file 12974_2021_2373_MOESM2_ESM.pdf]

A

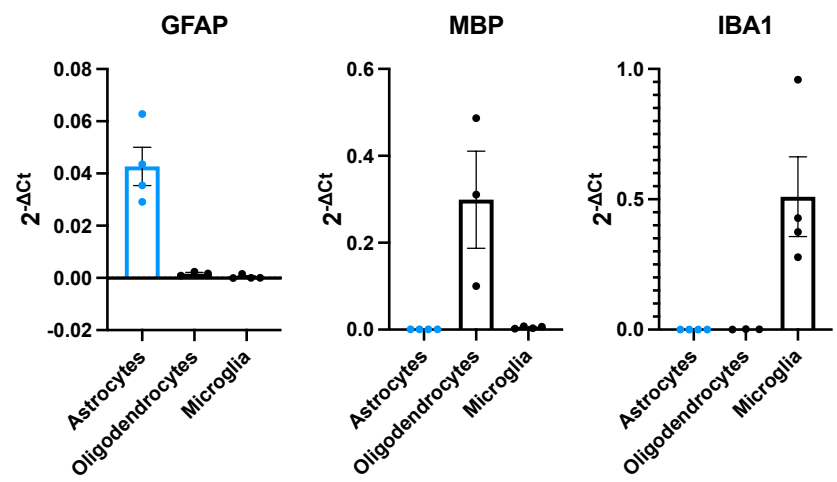

B

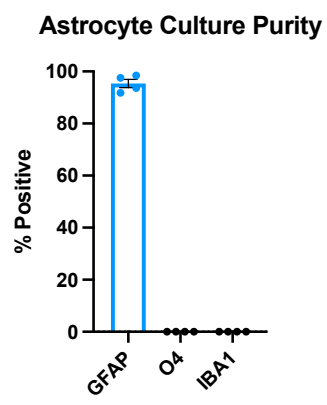

C

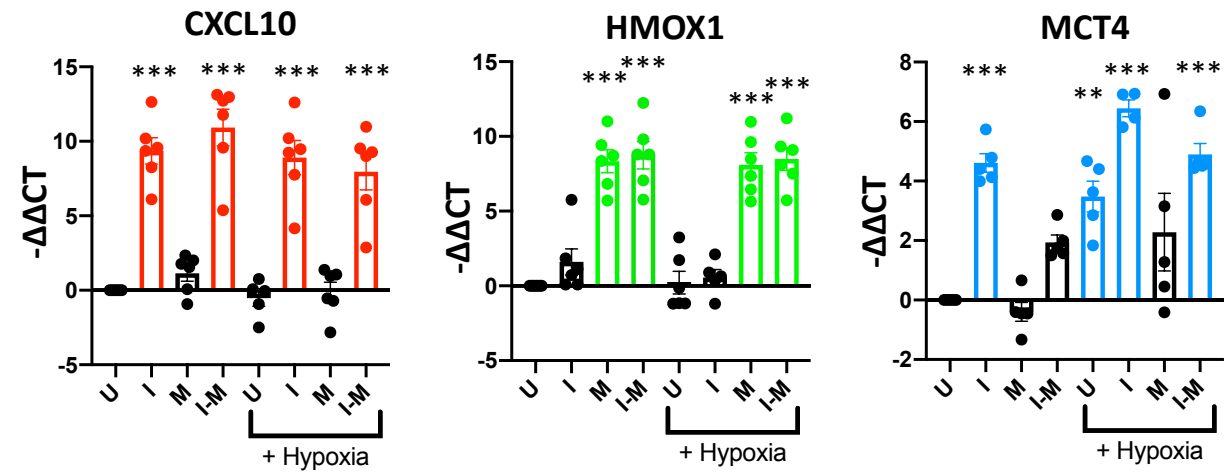

D

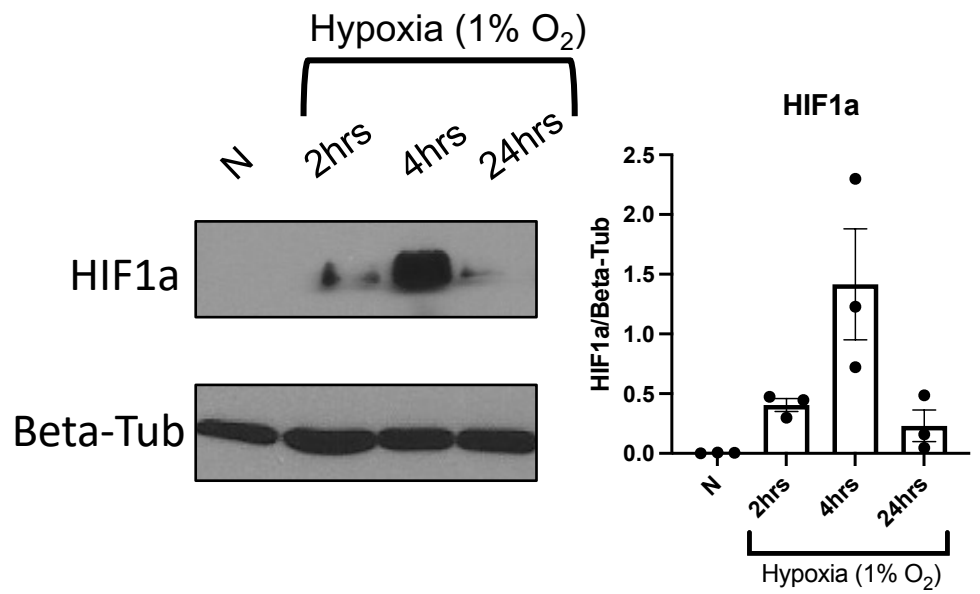

E

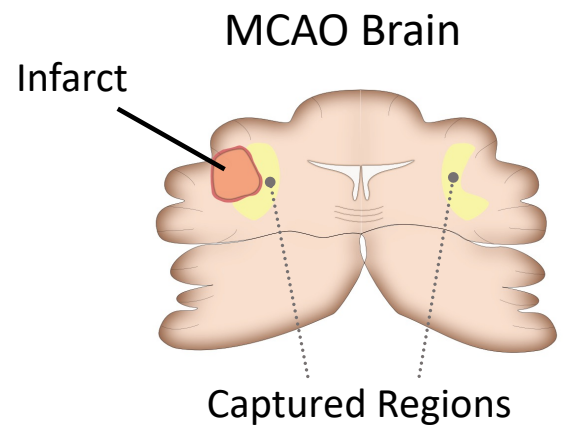

F

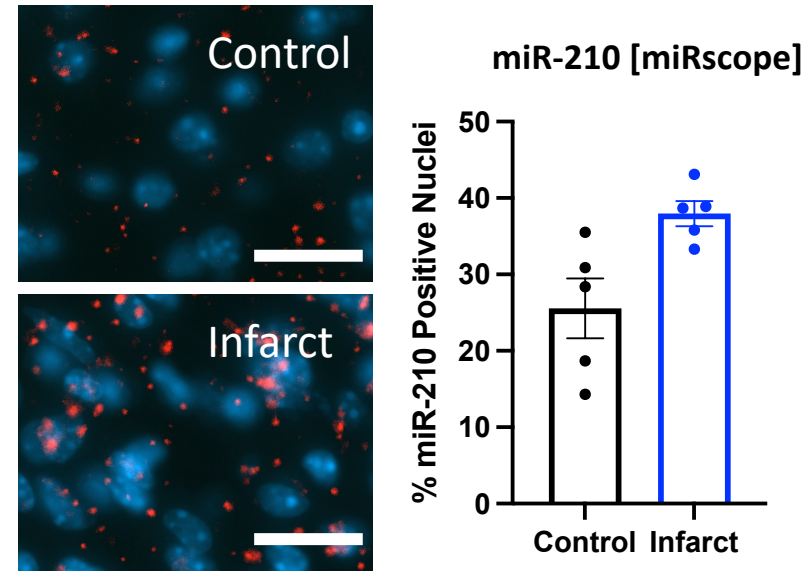

Supplement: Supplementary file 3 — Additional file 3: Figure S2. Primary human fetal astrocytes respond to in vitro stresses, and mice upregulate miR-210 around ischemic lesions. (A) Primary human astrocytes, oligodendrocytes, and microglia were separately cultured and analyzed for expression of canonically expressed genes of glial fibrillary acidic protein (GFAP), myelin basic protein (MBP) and ionized calcium binding adaptor molecule 1 (IBA1) by RT-qPCR. n = 3–4. (B) Primary human fetal astrocytes were immunostained with anti-GFAP, anti-O4, and anti-PU.1 antibodies. A CX7 automated microscope was used to quantify the percent positive astrocytes for each marker, n = 4. (C) Primary human astrocytes were treated in inflammatory (“I”) metabolic (“M”) or hypoxic (“H”) stress conditions induced by IL1b, glucose-free media, or a 1% oxygen chamber for 24 h compared to untreated control. RT-qPCR assessment of CXCL10, HMOX1, and MCT4 expression was measured to confirm the astrocytic response to inflammatory, metabolic, and hypoxic stress conditions, respectively, n = 4–6. (D) HeLa cells were left in normoxic (“N”) conditions for 48 h or were treated in 1% O2 (hypoxia, H) for the noted time. After 2–48 h, the cells were removed and were immediately lysed with RIPA buffer for subsequent Western blot. n = 3. (E) Graphical representation of the area investigated for miR-210 expression using miRscope. miR-210 was quantified in the highlighted region around the infarct site (left), and in the respectively highlighted region on the contralateral side of the brain (right). 300–500 cells were counted per brain section. (F) Representative images and quantification of microRNAscope (miRscope) of miR-210 around MCAO lesions (labeled as infarct) or on the contralateral side of the brain (labeled as control). Scale bar = 20 µm. Quantification was performed by manual counting of the number of nuclei positive for one or more red puncta. Red dots show miR-210 expression, and DAPI (blue) was used as a counterstain. Each dot i [file 12974_2021_2373_MOESM3_ESM.pdf]

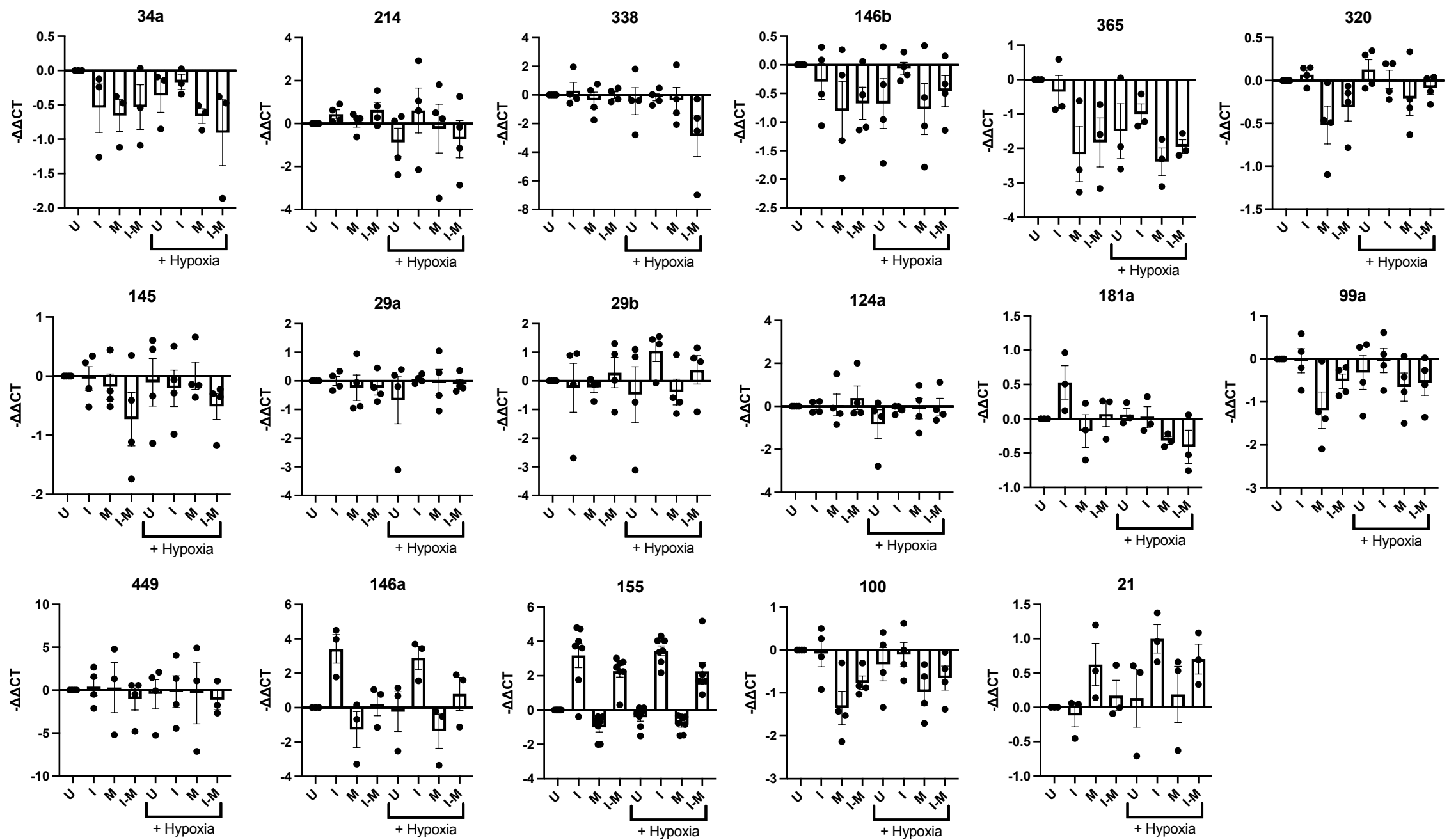

Supplement: Supplementary file 4 — Additional file 4: Figure S3. microRNAs are differentially expressed in primary human astrocytes undergoing different stresses. (A) Primary human astrocytes, oligodendrocytes, and microglia were separately cultured and analyzed for expression of canonically expressed genes of glial fibrillary acidic protein (GFAP), myelin basic protein (MBP) and ionized calcium binding adaptor molecule 1 (IBA1) by RT-qPCR. n = 3–4. (B) Primary human fetal astrocytes were immunostained with anti-GFAP, anti-O4, and anti-PU.1 antibodies. A CX7 automated microscope was used to quantify the percent positive astrocytes for each marker, n = 4. (C) Primary human astrocytes were treated in inflammatory (“I”) metabolic (“M”) or hypoxic (“H”) stress conditions induced by IL1b, glucose-free media, or a 1% oxygen chamber for 24 h compared to untreated control. RT-qPCR assessment of CXCL10, HMOX1, and MCT4 expression was measured to confirm the astrocytic response to inflammatory, metabolic, and hypoxic stress conditions, respectively, n = 4–6. (D) HeLa cells were left in normoxic (“N”) conditions for 48 h or were treated in 1% O2 (hypoxia, H) for the noted time period. After 2–48 h, the cells were removed and were immediately lysed with RIPA buffer for subsequent Western blot. n = 3. [file 12974_2021_2373_MOESM4_ESM.pdf]

A

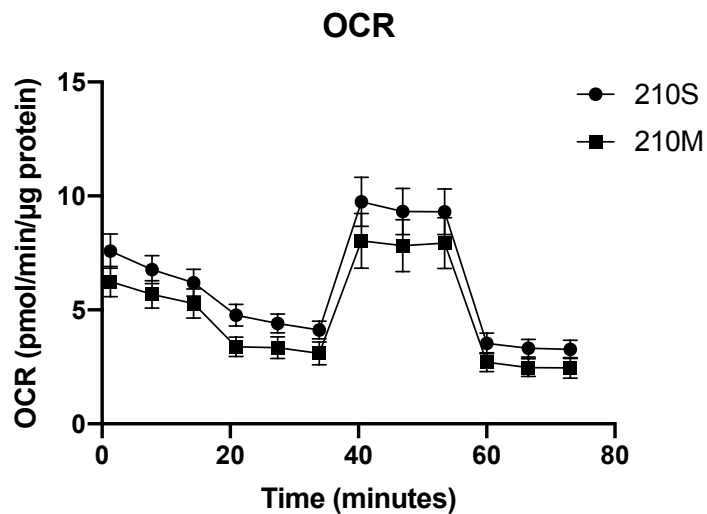

B

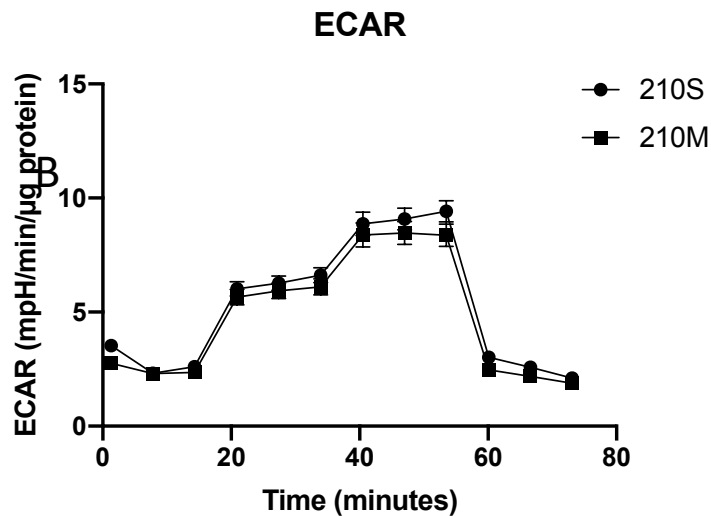

C

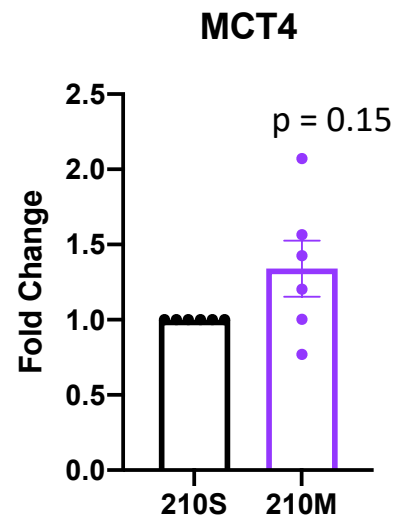

D

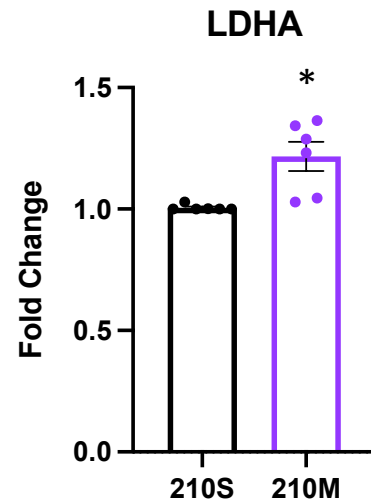

Supplement: Supplementary file 5 — Additional file 5: Figure S4. Astrocytic Metabolism and MCT4 Protein Expression is unchanged after 24- and 48-h 210M transfection, respectively. (A) Oxygen Consumption Rate (OCR) was measured in primary human astrocytes 24-h after transfection of 210S or 210M. n = 4 with 10 technical replicates for OCR experiments. (B) Extracellular Acidification Rate (ECAR) was measured in primary human astrocytes 24-h after transfection of 210S or 210M. n = 4 with 20 technical replicates for ECAR experiments. (C) Protein expression of MCT4 and Beta Tubulin (Beta-Tub) were measured by western blot in six separate human samples. Data is graphed as the fold change of MCT4 expression normalized to Beta-Tubulin expression and relative to the expression of 210S. For (C), each dot represents a separate human sample. (D) RT-qPCR assessment of LDHA in 210M-transfected cells relative to 210S-transfected cells 48 h after transfection. Mean ± SEM of 6 donors. Two-way ANOVA with Sidak’s correction was performed for Seahorse experiments, whereas t-test was used for MCT4 and LDHA expression. *p < 0.05. [file 12974_2021_2373_MOESM5_ESM.pdf]

**RELA**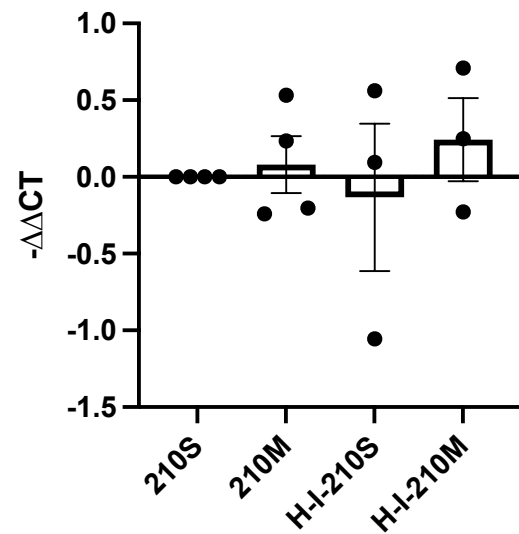**NFKB**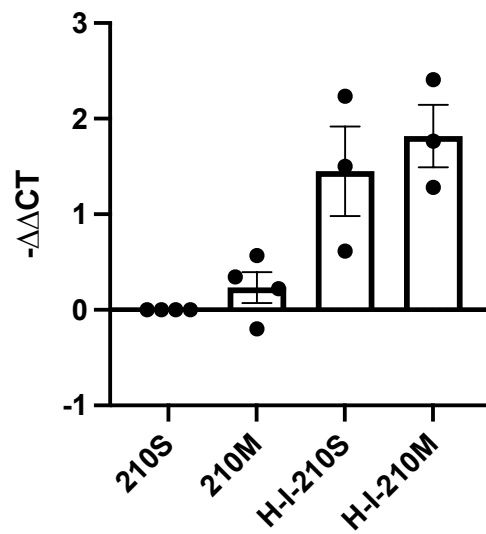**NOS2**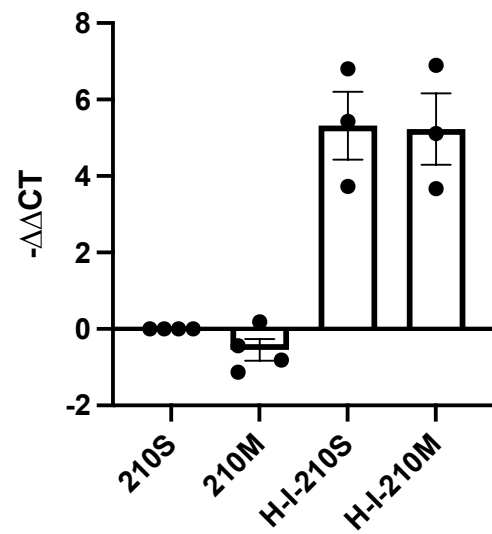**Nfe2l2**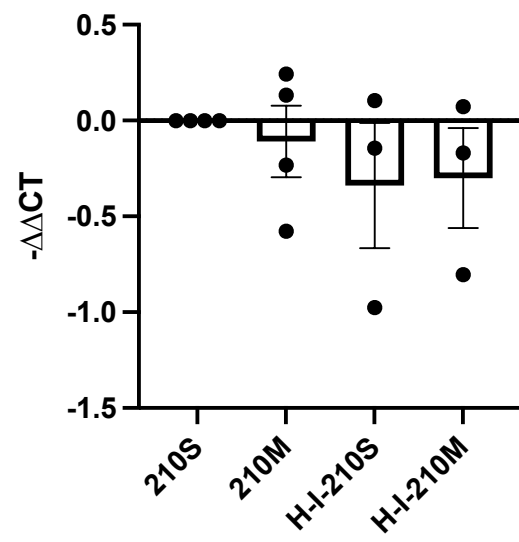**S100A10**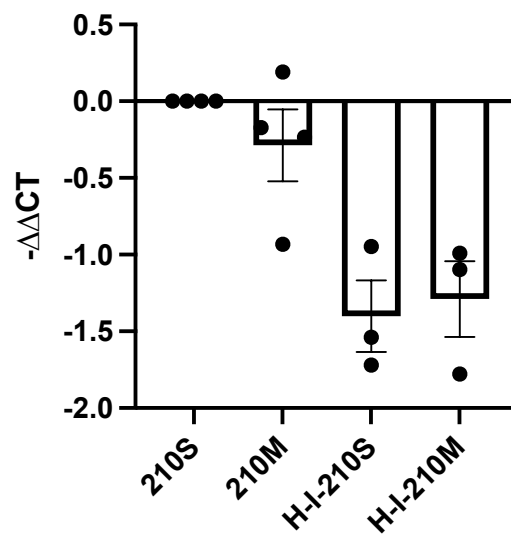

Supplement: Supplementary file 6 — Additional file 6: Figure S5. 210M does not affect astrocytic expression of select pro- or anti-inflammatory genes. Select genes that have previously been identified to promote or antagonize inflammation in astrocytes were measured by RT-qPCR in primary human fetal astrocytes. Cells were transfected with 210S or 210M and left otherwise untreated or treated with hypoxia (H) and inflammation (I) using 1% oxygen and IL1b, respectively. n = 3–4, data is graphed as mean ± SEM and was analyzed using one-way ANOVA with Sidak’s multiple comparison test. [file 12974_2021_2373_MOESM6_ESM.pdf]
